# Supplementary material for: Outer retinal features in OCT predict visual recovery after primary macula-involving retinal detachment repair
Source: PLoS One. 2022 May 5;17(5):e0268028. doi: 10.1371/journal.pone.0268028 (PMC9070941; doi:10.1371/journal.pone.0268028)
Supplement: S1 Table — Descriptive statistics of visual acuity (VA; measured in LogMAR) by subgroups with or without central foveal detachment in pars plana vitrectomy for rhegmatogenous retinal detachment with macular involvement. (DOCX) [file pone.0268028.s001.docx]

**S1 Table. Descriptive statistics of visual acuity by subgroups.**

Descriptive statistics of visual acuity (VA; measured in LogMAR) by subgroups with or without central foveal detachment in pars plana vitrectomy for rhegmatogenous retinal detachment with macular involvement.

| **Fovea ON** |  | |  | |  | |  | | |  | |  | | |  |  |
| --- | --- | --- | --- | --- | --- | --- | --- | --- | --- | --- | --- | --- | --- | --- | --- | --- |
| timepoint | N | Min. | | Max. | | Mean | | SD | Median | | 25 perc | | 75 perc |  |  |  |
| baseline VA | 10 | 0 | | 0.6 | | 0.18 | | 0.19 | 0.15 | | 0 | | 0.3 |  |  |  |
| 6 months VA | 10 | -0.1 | | 0.2 | | 0.06 | | 0.11 | 0 | | 0 | | 0.2 |  |  |  |
|  |  |  | |  | |  | |  |  | |  | |  |  |  |  |
| **Fovea OFF** |  |  | |  | |  | |  |  | |  | |  |  |  |  |
| timepoint | N | Min. | | Max. | | Mean | | SD | Median | | 25 perc | | 75 perc |  |  |  |
| baseline VA | 64 | 0.1 | | 2 | | 1.22 | | 0.56 | 1.4 | | 0.8 | | 1.5 |  |  |  |
| 6 months VA | 64 | -0.1 | | 1 | | 0.24 | | 0.21 | 0.2 | | 0.1 | | 0.3 |  |  |  |

Legend: 25 perc = 25th percentiles of the data, 75 perc = 75th percentiles of the data, IQR = interquartile range, NA = not applicable, n = numbers, SD = standard deviation, VA = visual acuity
