# Supplementary material for: Outer retinal features in OCT predict visual recovery after primary macula-involving retinal detachment repair
Source: PLoS One. 2022 May 5;17(5):e0268028. doi: 10.1371/journal.pone.0268028 (PMC9070941; doi:10.1371/journal.pone.0268028)
Supplement: S2 Table — Descriptive statistics of visual acuity (VA) and reading acuity (VA) by subgroups with or without ILM-peeling in pars plana vitrectomy for rhegmatogenous retinal detachment with macular involvement. (DOCX) [file pone.0268028.s002.docx]

**S2 Table. Descriptive statistics of visual and reading acuity by subgroups.**

Descriptive statistics of visual acuity (VA) and reading acuity (VA) by subgroups with or without ILM-peeling in pars plana vitrectomy for rhegmatogenous retinal detachment with macular involvement.

|  | **Distance visual acuity (VA) LogMAR** | | | | | | | | | | | | | | |
| --- | --- | --- | --- | --- | --- | --- | --- | --- | --- | --- | --- | --- | --- | --- | --- |
|  | Baseline | | | | | 1 month | | | | | 6 months | | | | |
| Group | mean | sd | median | IQR | n | mean | sd | median | IQR | n | mean | sd | median | IQR | n |
| peeling | 1.12 | 0.6 | 1.35 | 0.6 - 1.5 | 68 | 0.36 | 0.25 | 0.3 | 0.2 - 0.4 | 68 | 0.23 | 0.2 | 0.2 | 0.1 - 0.3 | 68 |
| no-peeling | 0.44 | 0.72 | 0.2 | 0.08 - 0.95 | 6 | 0.13 | 0.1 | 0.1 | 0.08 - 0.23 | 6 | 0.1 | 0.22 | 0 | -0.31 | 6 |
| pooled | 1.08 | 0.63 | 1.2 | 0.6 - 1.5 |  | 0.34 | 0.25 | 0.3 | 0.2 - 0.4 |  | 0.22 | 0.21 | 0.2 | 0.1 - 0.3 |  |
|  |  |  |  |  |  |  |  |  |  |  |  |  |  |  |  |
|  | **Reading acuity (RA) LogRAD** | | | | | | | | | | | | | | |
|  | Baseline | | | | | 1 month | | | | | 6 months | | | | |
| Group | mean | sd | median | IQR | n | mean | sd | median | IQR | n | mean | sd | median | IQR | n |
| peeling | 0.53 | 0.28 | 0.6 | 0.3 - 0.75 | 9 | 0.49 | 0.2 | 0.5 | 0.4 - 0.6 | 28 | 0.39 | 0.27 | 0.4 | 0.2 - 0.58 | 28 |
| no-peeling | 0.15 | 0.21 | 0.15 | 0 - 0.3 | 2 | 0.1 | NA | NA | NA | 1 | 0.05 | 0.07 | 0.05 | 0 - 0.1 | 2 |
| pooled | 0.46 | 0.3 | 0.3 | 0.3 - 0.6 | 11 | 0.48 | 0.21 | 0.5 | 0.4 - 0.6 | 29 | 0.37 | 0.28 | 0.35 | 0.1 - 0.53 | 30 |

Legend: IQR = interquartile range, NA = not applicable, n = numbers, RA = reading acuity, sd = standard deviation, VA = visual acuity
