# Supplementary material for: Outer retinal features in OCT predict visual recovery after primary macula-involving retinal detachment repair
Source: PLoS One. 2022 May 5;17(5):e0268028. doi: 10.1371/journal.pone.0268028 (PMC9070941; doi:10.1371/journal.pone.0268028)
Supplement: S3 Table — (DOCX) [file pone.0268028.s003.docx]

**S3 Tables. Detailed description of multiple regression models with correlations and prognostic factors 1 month after retinal detachment surgery with regard to visual function after 6 months.**

In a total of four multiple regression models, we included grading of ELM and EZ after 1 month, with and without VA and RA after 1 month, as predictors for VA and RA after 6 months (Table 1). The model summary indicates independence of observations, as assessed by the Durbin-Watson statistic (between 1.43 and 1.73). Inspection of the results supported the assumption of a linear relationship, no outliers, normal distributions of the residuals, and homoscedasticity. These results are presented in the main manuscript.

Here we present our further regression models including inner and outer photoreceptor segment height after 1 month, foveal detachment, foveal detachment height, retinal segmentation and undulation, as well as a correction for age and gender. In these models, we did not find a relevant influence of these factors on our main outcomes.

Regression model analyzing the effect of Inner (IS) and Outer (OS) Photoreceptor segment after one month on visual (VA) and reading (RA) acuity after six months.

|  | *β* | *t* | *p* | *F* | *df* | *p* | *adj. R^2^* | |
| --- | --- | --- | --- | --- | --- | --- | --- | --- |
| **Dependent variable:  Visual acuity 6 months after surgery** | | | | | | | | |
| Model 5 |  |  |  | 3.933 | 2 | 0.024 | 0.074 | |
| IS after 1 month | -0.005 | -2.14 | 0.036* |  |  |  |  | |
| OS after 1 month | -0.002 | -0.98 | 0.330 |  |  |  |  | |
|  |  |  |  |  |  |  |  | |
|  |  |  |  |  |  |  |  | |
| **Dependent variable:  Reading acuity 6 months after surgery** | | | | | | | |  |
| Model 6 |  |  |  | 3.616 | 2 | 0.041 | 0.153 | |
| IS after 1 month | -0.0043 | -0.887 | 0.383 |  |  |  |  | |
| OS after 1 month | -0.0074 | -2.174 | 0.039* |  |  |  |  | |
|  |  |  |  |  |  |  |  | |

Regression model analyzing the effect of a presence of foveal detachment (FD), the foveal detachment height (DH), and retinal separation (SEP) and undulation (UND) at baseline before surgery on visual (VA) and reading (RA) acuity after six months

|  | *β* | *t* | *p* | *F* | *df* | *p* | *adj. R^2^* | |
| --- | --- | --- | --- | --- | --- | --- | --- | --- |
| **Dependent variable:  Visual acuity 6 months after surgery** | | | | | | | | |
|  |  |  |  |  |  |  |  | |
| Model 7 |  |  |  | 2.018 | 4 | 0.11 | 0.087 | |
| FD at baseline | 0.188 | 2.028 | 0.0494* |  |  |  |  | |
| DH at baseline | 0.00006 | 0.533 | 0.597 |  |  |  |  | |
| SEP at baseline | -0.167 | -1.819 | 0.077 |  |  |  |  | |
| UND at baseline | 0.036 | 0.369 | 0.714 |  |  |  |  | |
|  |  |  |  |  |  |  |  | |
| **Dependent variable:  Reading acuity 6 months after surgery** | | | | | | | |  |
|  |  |  |  |  |  |  |  | |
| Model 8 |  |  |  | 2.70 | 4 | 0.087 | 0.312 | |
| FD at baseline | 0.100 | 0.504 | 0.624 |  |  |  |  | |
| DH at baseline | 0.0004 | 1.910 | 0.083 |  |  |  |  | |
| SEP at baseline | -0.486 | -2.019 | 0.069 |  |  |  |  | |
| UND at baseline | 0.436 | 1.204 | 0.254 |  |  |  |  | |

Regression model, controlling for age and sex, analyzing the effect of gradings of external limiting membrane (ELM) and ellipsoid zone (EZ) after one month in Model 1, and with the inclusion of visual (VA) and reading (RA) acuity after one month in Model 2, on visual (VA) and reading (RA) acuity after six months. This model is the same as in the main manuscript, extended by the correction for age and sex. * indicates p<0.05; ** indicates p<0.01

|  | *β* | *t* | *p* | *F* | *df* | *p* | *adj. R^2^* | |
| --- | --- | --- | --- | --- | --- | --- | --- | --- |
| **Dependent variable:  Visual acuity 6 months after surgery** | | | | | | | | |
| Model 9 |  |  |  | 2.557 | 4 | 0.046 | 0.079 | |
| age | 0.0033 | 1.374 | 0.174 |  |  |  |  | |
| sex | -0.008 | -0.159 | 0.874 |  |  |  |  | |
| ELM after 1 month | 0.115 | 2.654 | 0.0099** |  |  |  |  | |
| EZ after 1 month | -0.044 | -0.874 | 0.385 |  |  |  |  | |
|  |  |  |  |  |  |  |  | |
| Model 10 |  |  |  | 8.379 | 6 | 8.44e-05 | 0.61 | |
| age | 0.004 | 1.464 | 0.157 |  |  |  |  | |
| sex | -0.050 | -0.844 | 0.408 |  |  |  |  | |
| ELM after 1 month | -0.004 | -0.076 | 0.940 |  |  |  |  | |
| EZ after 1 month | 0.125 | 1.973 | 0.061 |  |  |  |  | |
| VA after 1 month | 0.492 | 2.671 | 0.014* |  |  |  |  | |
| RA after 1 month | 0.138 | 0.635 | 0.532 |  |  |  |  | |
|  |  |  |  |  |  |  |  | |
| **Dependent variable:  Reading acuity 6 months after surgery** | | | | | | | |  |
| Model 11 |  |  |  | 3.406 | 4 |  | 0.249 | |
| age | 0.0038 | 0.816 | 0.422 |  |  |  |  | |
| sex | 0.017 | 0.177 | 0.861 |  |  |  |  | |
| ELM after 1 month | 0.263 | 3.326 | 0.0027** |  |  |  |  | |
| EZ after 1 month | -0.064 | -0.610 | 0.547 |  |  |  |  | |
|  |  |  |  |  |  |  |  | |
| Model 12 |  |  |  | 3.546 | 6 | 0.017 | 0.389 | |
| age | 0.003 | 0.594 | 0.560 |  |  |  |  | |
| sex | 0.022 | 0.239 | 0.813 |  |  |  |  | |
| ELM after 1 month | 0.088 | 1.013 | 0.324 |  |  |  |  | |
| EZ after 1 month | 0.007 | 0.072 | 0.944 |  |  |  |  | |
| VA after 1 month | 0.281 | 0.880 | 0.390 |  |  |  |  | |
| RA after 1 month | 0.366 | 1.009 | 0.326 |  |  |  |  | |

Regression model, controlling for internal limiting membrane (ILM) peeling (with or without), analyzing the effect of gradings of external limiting membrane (ELM) and ellipsoid zone (EZ) after one month in Model 1, and with the inclusion of visual (VA) and reading (RA) acuity after one month in Model 2, on visual (VA) and reading (RA) acuity after six months. * indicates p<0.05; ** indicates p<0.01

|  | *β* | *t* | *p* | *F* | *df* | *p* | *adj. R^2^* | |
| --- | --- | --- | --- | --- | --- | --- | --- | --- |
| **Dependent variable:  Visual acuity 6 months after surgery** | | | | | | | | |
| Model 13 |  |  |  | 3.001 | 3 | 0.036 | 0.076 | |
| ILM-peeling | 0.073 | 0.836 | 0.406 |  |  |  |  | |
| ELM after 1 month | 0.098 | 2.294 | 0.025* |  |  |  |  | |
| EZ after 1 month | -0.027 | -0.560 | 0.577 |  |  |  |  | |
|  |  |  |  |  |  |  |  | |
| Model 14 |  |  |  | 8.574 | 5 | 0.0001 | 0.575 | |
| ILM-peeling | 0.020 | 0.126 | 0.901 |  |  |  |  | |
| ELM after 1 month | -0.034 | -0.615 | 0.545 |  |  |  |  | |
| EZ after 1 month | 0.141 | 2.155 | 0.042* |  |  |  |  | |
| VA after 1 month | 0.465 | 2.525 | 0.019* |  |  |  |  | |
| RA after 1 month | 0.217 | 1.021 | 0.318 |  |  |  |  | |
|  |  |  |  |  |  |  |  | |
| **Dependent variable:  Reading acuity 6 months after surgery** | | | | | | | |  |
| Model 15 |  |  |  | 5.284 | 3 | 0.0056 | 0.307 | |
| ILM-peeling | 0.233 | 1.348 | 0.189 |  |  |  |  | |
| ELM after 1 month | 0.241 | 3.159 | 0.004** |  |  |  |  | |
| EZ after 1 month | -0.067 | -0.668 | 0.510 |  |  |  |  | |
|  |  |  |  |  |  |  |  | |
| Model 16 |  |  |  | 4.371 | 5 | 0.008 | 0.413 | |
| ILM-peeling | 0.070 | 0.322 | 0.751 |  |  |  |  | |
| ELM after 1 month | 0.091 | 1.099 | 0.285 |  |  |  |  | |
| EZ after 1 month | -0.005 | -0.05 | 0.961 |  |  |  |  | |
| VA after 1 month | 0.208 | 0.744 | 0.466 |  |  |  |  | |
| RA after 1 month | 0.452 | 1.452 | 0.163 |  |  |  |  | |

Regression model, controlling for foveal detachment (FD), analyzing the effect of gradings of external limiting membrane (ELM) and ellipsoid zone (EZ) after one month in Model 1, and with the inclusion of visual (VA) and reading (RA) acuity after one month in Model 2, on visual (VA) and reading (RA) acuity after six months. * indicates p<0.05; ** indicates p<0.01

|  | *β* | *t* | *p* | *F* | *df* | *p* | *adj. R^2^* | |
| --- | --- | --- | --- | --- | --- | --- | --- | --- |
| **Dependent variable:  Visual acuity 6 months after surgery** | | | | | | | | |
| Model 1 |  |  |  | 4.63 | 3 | 0.0052 | 0.13 | |
| FD | 0.149 | 2.252 | 0.027* |  |  |  |  | |
| ELM after 1 month | 0.089 | 2.130 | 0.037* |  |  |  |  | |
| EZ after 1 month | -0.021 | -0.454 | 0.651 |  |  |  |  | |
|  |  |  |  |  |  |  |  | |
| Model 2 |  |  |  | 8.722 | 5 | 9.438e-05 | 0.58 | |
| FD | 0.06 | 0.523 | 0.606 |  |  |  |  | |
| ELM after 1 month | -0.036 | -0.674 | 0.507 |  |  |  |  | |
| EZ after 1 month | 0.143 | 2.200 | 0.038* |  |  |  |  | |
| VA after 1 month | 0.473 | 2.579 | 0.017* |  |  |  |  | |
| RA after 1 month | 0.185 | 0.862 | 0.397 |  |  |  |  | |
|  |  |  |  |  |  |  |  | |
| **Dependent variable:  Reading acuity 6 months after surgery** | | | | | | | |  |
| Model 1 |  |  |  | 7.075 | 3 | 0.0012 | 0.386 | |
| FD | 0.276 | 2.321 | 0.028* |  |  |  |  | |
| ELM after 1 month | 0.228 | 3.162 | 0.004** |  |  |  |  | |
| EZ after 1 month | -0.044 | -0.460 | 0.649 |  |  |  |  | |
|  |  |  |  |  |  |  |  | |
| Model 2 |  |  |  | 4.571 | 5 | 0.0066 |  | |
| FD | 0.121 | 0.756 | 0.459 |  |  |  |  | |
| ELM after 1 month | 0.082 | 1.005 | 0.327 |  |  |  |  | |
| EZ after 1 month | 0.0009 | 0.010 | 0.992 |  |  |  |  | |
| VA after 1 month | 0.229 | 0.827 | 0.419 |  |  |  |  | |
| RA after 1 month | 0.394 | 1.252 | 0.226 |  |  |  |  | |
