# Supplementary material for: Outer retinal features in OCT predict visual recovery after primary macula-involving retinal detachment repair
Source: PLoS One. 2022 May 5;17(5):e0268028. doi: 10.1371/journal.pone.0268028 (PMC9070941; doi:10.1371/journal.pone.0268028)
Supplement: Attachment — Submitted filename: Response to Reviewers.docx [file pone.0268028.s005.docx]

*PLOS ONE* Editorial Board

Academic Editor

Akram Belghith

**Response to Reviewers**

**Outer retinal features in OCT predict visual recovery after primary macula-involving retinal detachment repair**

Bern, March 6, 2022

Dear Akram Belghith, dear Reviewer,

Thank you for your kind appreciation of the scientific and clinical value of our study in the still debated topic of retinal detachments with macular involvement.

- As discussed in our manuscript and justifiably recalled by the reviewer, most patients underwent ILM peeling due to personal preference of the surgeon, which may not be universally accepted standard of care. The limited generalizability for patients without ILM peeling was added in the revised discussion.
- Also highlighted by the reviewer ILM peeling may cause considerable changes to the retinal layers, with possibly longitudinal retinal layer thickness changes. The concern is addressed with an additional reference (Hisatomi et al, Retina 2018 Mar;38(3):471-479) in the revised discussion.
- The previously mentioned data of retinal layer changes after macula off retinal detachment repair has been formulated more explicitly and the suggested reference (Invest Ophthalmol Vis Sci. 2014 Sep 4;55(10):6575-9.) was added to the previously quoted literature.

We believe that we have answered all the criticized points sufficiently and implemented them in the revised manuscript, highlighted in the track-changed version. Also, the additional style guides have been implemented in the revised manuscript.

Thank you for considering the revised manuscript of our study for publication in PLOS one.

Sincerely yours,

Christof Hänsli
